# Family Meal Frequency and Association with Household Food Availability in United States Multi-Person Households: National Health and Nutrition Examination Survey 2007-2010

**DOI:** 10.1371/journal.pone.0144330

**Published:** 2015-12-04

**Authors:** Sarah L. Newman, Rachel Tumin, Rebecca Andridge, Sarah E. Anderson

**Affiliations:** 1 Division of Epidemiology, College of Public Health, The Ohio State University, Columbus, Ohio, United States of America; 2 Division of Biostatistics, College of Public Health, The Ohio State University, Columbus, Ohio, United States of America; Tufts University, UNITED STATES

## Abstract

**Objective:**

Family meals are associated with a healthier diet among children and adolescents, but how family meal frequency varies in the U.S. population overall by household food availability and sociodemographic characteristics is not well characterized.

**Design:**

The U.S. National Health and Nutrition Examination Survey 2007–2010 assessed the frequency of family meals eaten at home in the past week and the household availability of fruits, dark green vegetables, salty snacks, and sugar-sweetened beverages.

**Setting:**

Computer-assisted face-to-face interviews with a selected adult (≥18 years) who owned or rented the home (i.e., the household reference person).

**Subjects:**

We analyzed information on family meal frequency for 18,031 participants living in multi-person households in relation to sociodemographic characteristics and food availability.

**Results:**

Among the U.S. population living in households of two or more individuals, the prevalence (95% confidence interval) of having 0–2, 3–6 and ≥7 family meals/week was 18.0% (16.6–19.3), 32.4% (31.0–33.9), and 49.6% (47.8–51.4), respectively. Greater household availability of fruits and dark green vegetables and less availability of salty snacks and sugar-sweetened beverages was associated with more frequent family meals. Family meals were more prevalent in low-income households and those in which the reference person was ≥65 years, married, or had less than high school education.

**Conclusions:**

About half of the US population living in households of 2 or more people shares meals frequently with their family at home. Family meal frequency was positively associated with a healthier pattern of household food availability.

## Introduction

Although not without potential difficulties [1,2], family meals are increasingly promoted as a strategy for improving public health and preventing obesity [3,4,5]. Research on family meals has primarily focused on children and adolescents, but a wider examination of the prevalence, correlates, and benefits of family meals for individuals across the life course is beginning [4]. At present, research on family meals in adults, particularly adults who are not parents of minor children, is limited [4,6,7]. In a large representative sample of Ohio adults surveyed in 2012, differences were found in family meal frequency relative to sociodemographic characteristics such as marital status, race/ethnicity, employment, and age, but little impact on family meal frequency was found for having minor children in the household [8]. Information about the prevalence and correlates of family meals among the U.S. population has been previously reported [6,9]. However, those estimates were based on telephone surveys of fewer than 1000 adults.

Among children and adolescents, substantial evidence suggests that frequent family meals are associated with a more nutritious pattern of dietary intake [4,10,11]. Longitudinal analyses of Project Eating Among Teens (EAT) have shown that the frequency of family meals in adolescence is related to subsequent diet quality [12,13], and lower risk for obesity [14]. Project EAT studied more than 2000 Minnesota youth from 1998–1999 (when participants were a mean age of 15 years) to 2008–2009 when they ranged in age from 19 to 31 years (mean 25 years); frequency of family meals in adolescence was positively correlated with eating with others in young adulthood which in turn was linked to greater reported intake of nutrient dense foods such as fruits and vegetables, particularly for females [13]. Compared to having no family meals as adolescents, any frequency of family meals was associated with reduced risk for overweight and obesity in young adulthood [14]. Among more than 3500 parents of adolescents studied in Minnesota in 2009–10, Berge and colleagues found a positive association between family meal frequency and consumption of fruits and vegetables for mothers and fathers [7]; they also examined the association between family meal frequency and fast food consumption and found that for fathers, but not for mothers, frequent family meals were associated with less consumption of fast food [7]. The extent to which frequency of family meals in the U.S. population is related to the nutritional quality of food available in the household has not been previously examined. Understanding patterns and correlates of family meal practices at a population level is important for framing health communication strategies.

Our objective in this report was to add to the growing literature on the epidemiology of family meals in the U.S. population. We analyzed nationally representative data on U.S. multi-person households to describe the prevalence of family meals relative to sociodemographic characteristics and the healthfulness of foods available in the household. Based on research on children and adolescents, we hypothesized that households with greater availability of fruits and vegetables and lower availability of sugar-sweetened beverages and salty snacks would report more frequent family meals.

## Methods

### Study population

We analyzed data on family meals and household food availability collected during the 2007–08 and 2009–10 cycles of the National Health and Nutrition Examination Survey (NHANES). NHANES is conducted by the National Center for Health Statistics (NCHS) and each two-year cycle is designed to be representative of the U.S. civilian non-institutionalized population. NCHS uses a multi-stage, stratified, clustered probability sampling design that includes an oversample of some population subgroups. Participants in NHANES are interviewed and assessed by trained staff using standardized procedures. Detailed information about the design and operations of the survey are published [15,16]. All NHANES participants provided informed consent and procedures were reviewed and approved by the NCHS Research Ethics Review Board [15]. We analyzed the publicly available data which are deidentified and not considered human subjects research by the IRB at our institution.

In each household, NHANES identifies an adult (≥18 years) who owns or rents the residence as the household reference person, and this individual reports on characteristics of the household. During the 2007–08 and 2009–10 cycles of NHANES, but not in prior or subsequent years, a series of questions on family meals and household food availability were included as part of the in-home, face-to-face interview with the household reference person. Our analyses include all NHANES participants living in households with two or more people. Single-person households were not asked about family meals.

### Variables

#### Frequency of family meals

The household reference person was asked, "During the **past 7 days**, how many meals did all or **most of your family** sit down and eat together at home?" Interviewers were instructed to emphasize the bolded words, and if more than 21 meals in the past 7 days were reported, to verify that the “family eats at home more than 3 meals per day.” NHANES did not ask about family meals eaten in settings other than the home (e.g., restaurants). We categorized family meal frequency as 0–2 meals per week (few), 3–6 meals per week (some), and 7 or more meals per week (many) to create a 3-level variable that allowed for the greatest comparability to other published reports [7,9].

#### Household food availability

The household reference person also reported the availability of fruits, dark green vegetables, salty snacks and sugar-sweetened beverages in the home [17]. Questions were of the form, “How often does your family have **fruits** available at home? This includes fresh, dried, canned and frozen fruits. Would you say always, most of the time, sometimes, rarely, or never?” The five-level response options were the same for each of the questions and were provided on a card for reference [18]. The question about dark green vegetables read, “How often does your family have any of these **dark green vegetables** available at home? This includes fresh, dried, canned and frozen vegetables.” A card listing the following dark green vegetables was provided for the respondent to reference: bok choy, broccoli, collard greens, dark green leafy lettuce, kale, mesclun, mustard greens, romaine lettuce, turnip greens, spinach, watercress [18]. Availability of salty snacks was assessed by asking, "How often does your family have **salty snacks** such as chips and crackers available at home? Do not include nuts.” Availability of sugar-sweetened beverages was assessed with the question, “How often does your family have **soft drinks, fruit-flavored drinks, or fruit punch** available at home? Please do not include diet drinks, 100 percent juice or sports drinks.”

For each of these four types of foods we created binary measures of "healthier" and "less healthy" household food availability. For fruits and dark green vegetables the healthier responses were "always" or "most of the time," while for sugar-sweetened beverages and salty snacks the healthier responses were "rarely" or "never." In each binary measure, we coded a healthier response as 1 and a less healthy response as 0. We then summed these four binary variables to create an aggregate household healthy food availability score. This ordinal variable ranged from 0 (indicating low availability of fruits and dark green vegetables and high availability of sugar-sweetened beverages and salty snacks) to 4 (indicating high availability of fruits and dark green vegetables and low availability of sugar-sweetened beverages and salty snacks).

#### Household sociodemographic characteristics

The household reference person reported their age, marital status, education level, and country of birth. We created categorical variables for age (18–25 years, 26–45 years, 46–64 years, and ≥65 years), marital status (married or living with a partner, separated/divorced/widowed, and never married), education level (<9^th^ grade, 9^th^-11^th^ grade, high school graduate or GED equivalent, some college including Associate or technical degree, and Bachelor’s degree or higher), and country of birth (born in the U.S. or not). NHANES provides a continuous measure of the household’s income-to-poverty ratio based on household income and household size. We created a three-level categorical variable (<1.3, 1.3–3.5, >3.5); these cut points were used for comparability with other studies [19]. We categorized race/ethnicity as Hispanic (any race), non-Hispanic white, non-Hispanic black, or non-Hispanic other race (including multiple races) to facilitate understanding of the social determinants of health [20,21], and for comparability with the literature [8,9,19]. Although, the household reference person did not report on their own race or ethnicity, we inferred this information based on the race and ethnicity of the NHANES participant in the household.

### Analytic approach

We pooled data from the 2007–08 and 2009–10 cycles as recommended in the NHANES analytic guidelines [22], and included in our analyses all participants living in multi-person households. Of the 20,686 NHANES participants, we excluded 2,343 (11.3%) who lived alone. We excluded an additional 312 individuals (1.5%) missing information on family meals or household food availability. Thus, our analytic sample included 18,031 individuals, but due to missing data for some sociodemographic characteristics the sample size for the stratified analyses varied from 16,530 to 18,031. All analyses were conducted using SAS version 9.2 (Cary, NC). NHANES interview weights were applied such that estimates are representative of the U.S. civilian non-institutionalized population living in households of two or more people; variance estimates that account for the complex sample design were calculated with Taylor series linearization in the SAS Survey Procedures.

We estimated the proportion of the population that eats few family meals at home (0–2 meals per week), some family meals at home (3–6 meals per week) and many family meals at home (≥7 meals per week), as well as the associated 95% confidence intervals, overall and within levels of each sociodemographic characteristic. The Rao-Scott design-corrected chi-square was used to determine whether the frequency of family meals differed across levels of each sociodemographic characteristic. We considered a P value <0.05 as indicating statistical significance. No adjustment was made for multiple-comparisons.

We used cumulative logistic regression models to estimate odds ratios and 95% confidence intervals for having more frequent family meals in relation to home food availability. The outcome in these models was the 3-level frequency of family meals. The predictor, in separate models, was the binary fruit, dark green vegetable, salty snacks, or sugar-sweetened beverage variable with the less healthy response as the reference category. In the model in which the ordinal aggregate healthy food availability score was the predictor, the odds ratio calculated was for a 1 unit higher score. These models assume proportional odds [23] and the estimated odds ratio is appropriate for describing the comparison of 0–2 family meals per week to ≥3 family meals per week, as well as the comparison of ≤6 family meals per week to ≥7 family meals per week.

## Results

Among the U.S. population living in multi-person households, 18% ate few family meals at home (0–2 meals per week), approximately one-third (32.4%) ate 3 to 6 family meals at home per week, and half (49.6%) ate family meals frequently (7 or more meals per week). Table 1 presents the frequency of family meals in these three categories within strata of sociodemographic characteristics; frequency of family meals was statistically significantly associated with all of the characteristics examined (P<0.0001). Family meals were more frequent in households in which the household reference person was older. The prevalence of ≥7 family meals per week was 67.2% in households headed by someone ≥65 years and <50% for all younger households. Younger households were more likely to have ≤2 family meals per week.

**Table 1:** Family meal frequency in U.S. multi-person households by sociodemographic characteristics of the householder.

	Frequency of family meals at home in the past week				
		Percentage (95% CI)^b^			
	n^a^	0–2 meals/week	3–6 meals/week	≥7 meals/week	P value^c^
**Overall**	18031	18.0 (16.6, 19.3)	32.4 (31.0, 33.9)	49.6 (47.8, 51.4)	
**Age**					
18–25 years	1471	25.4 (19.1, 31.6)	32.2 (25.6, 38.8)	42.4 (37.2, 47.6)	<0.0001
26–45 years	9108	16.7 (15.1, 18.2)	33.8 (31.9, 35.7)	49.6 (47.4, 51.8)	
46–64 years	5119	20.6 (18.3, 22.9)	34.7 (32.3, 37.2)	44.7 (41.8, 47.5)	
≥65 years	2333	11.9 (9.0, 14.7)	20.9 (18.0, 23.8)	67.2 (63.9, 70.6)	
**Race/ethnicity** ^d^					
Hispanic, any race	6289	17.6 (15.5, 19.7)	24.1 (21.6, 26.5)	58.5 (55.3, 61.4)	<0.0001
Non-Hispanic white	7155	15.4 (13.6, 17.3)	34.9 (33.0, 36.8)	49.7 (47.7, 51.6)	
Non-Hispanic black	3595	34.0 (30.1, 37.9)	34.3 (31.4, 37.1)	31.7 (27.9, 35.5)	
Other race, non-Hispanic	992	14.4 (9.7, 19.2)	26.2 (20.3, 32.1)	59.4 (53.7, 65.0)	
**Education**					
<9^th^ grade	2148	17.6 (13.2, 22.0)	19.2 (15.8, 22.5)	63.2 (57.6, 68.9)	<0.0001
9^th^-11^th^ grade	3134	19.4 (15.6, 23.3)	25.1 (21.1, 29.1)	55.5 (51.4, 59.5)	
High School/GED	4322	21.0 (17.8, 24.2)	31.4 (27.9, 34.9)	47.6 (44.4, 50.9)	
Some college	4627	18.5 (15.5, 21.5)	34.2 (31.5, 36.9)	47.4 (44.4, 50.3)	
College graduate	3320	13.7 (11.4, 16.0)	38.3 (35.1, 41.6)	47.9 (44.6, 51.3)	
**Marital status**					
Married/living with partner	12935	14.9 (13.3, 16.5)	32.4 (30.4, 34.3)	52.8 (50.6, 54.9)	<0.0001
Divorced/separated/widowed	2749	27.6 (23.1, 32.1)	32.7 (28.4, 37.0)	39.7 (34.8, 44.6)	
Never married	1890	29.9 (24.1, 35.8)	32.8 (28.0, 37.5)	37.3 (32.4, 42.2)	
**Income to poverty ratio**					
<1.3	6290	19.7 (17.6, 21.7)	24.4 (21.8, 27.1)	55.9 (52.7, 59.1)	<0.0001
1.3–3.5	6042	18.3 (16.2, 20.5)	32.4 (29.4, 35.4)	49.3 (45.9, 52.6)	
>3.5	4198	16.5 (14.5, 18.5)	38.0 (35.2, 40.8)	45.5 (42.9, 48.2)	
**Country of birth**					
U.S.	12439	18.1 (16.6, 19.6)	34.6 (32.8, 36.5)	47.3 (45.2, 49.3)	<0.0001
Foreign born	5153	17.0 (15.1, 18.8)	23.0 (20.1, 26.0)	60.0 (56.2, 63.8)	

^a^ Unweighted sample n. Data were missing on education for 480, marital status for 457, income-to-poverty ratio for 1501, and country of birth for 439.

^b^ Percentages are weighted and 95% confidence intervals account for the complex sample design. Percentages may not total 100% due to rounding.

^c^ P-value for difference in groups from Rao-Scott design-corrected Chi-square.

^d^ Race/ethnicity of the sampled participant.

The distribution of family meals varied by race/ethnicity: Non-Hispanic blacks were equally likely to report 0–2 meals, 3–6 meals, or ≥7 family meals at home per week (34.0%, 34.3%, 31.7%, respectively) whereas, other racial/ethnic groups, particularly Hispanics, were more likely to report ≥7 family meals per week. Households headed by respondents who had not finished high school were more likely to have ≥7 family meals per week (63.2% and 55.5% for respondents with <9^th^ grade and 9^th^-11^th^ grade levels of education, respectively), but at higher levels of education the percentage reporting ≥7 family meals per week was ~47.5% regardless of educational attainment (Table 1).

Households that included partners who were married or living together had more frequent family meals than households headed by respondents who were divorced, separated, widowed, or never married. Nearly twice as many unmarried households had few (0–2) family meals per week compared to married/partnered households (Table 1). Increasing income-to-poverty ratio was negatively associated with reporting ≥7 family meals per week: 55.9%, 49.3%, and 45.5% of households with income-to-poverty ratios of <1.3, 1.3–3.5, and >3.5, respectively, ate ≥7 family meals per week. Households headed by someone born outside the U.S. were substantially more likely to report frequent family meals (Table 1).

The percentage of individuals in U.S. multi-person households who always, most of the time, sometimes, rarely, or never had fruits, dark green vegetables, salty snacks, and sugar-sweetened beverages available in the home is tabulated in Table 2. Households were most likely to always have fruits available (71.5%), whereas 58.5% of individuals lived in households in which dark green vegetables were always available. Fewer individuals lived in households in which salty snacks (45.2%) and sugar-sweetened beverages (42.8%) were always available (Table 2). Approximately 90% and 81% of individuals living in U.S. multi-person households met our definition of healthier household availability of fruits and dark green vegetables, respectively. Substantially fewer were classified as having healthy availability of salty snacks and sugar-sweetened beverages (11.7% and 26.0% rarely or never had salty snacks and sugar-sweetened beverages, respectively, available in the home) (Table 2). About 5% of individuals in U.S. multi-person households met our healthier availability definition for all four food categories (i.e., an aggregate healthy food availability score of 4), and a similar proportion (4.1%) did not meet our healthier availability definition for any of the food categories (i.e., aggregate score of 0) (Table 2).

**Table 2:** Home availability of fruits, dark green vegetables, salty snacks, and sugar-sweetened beverages for U.S. multi-person households: NHANES 2007–10.

Food category	Home availability	n^a^	Percentage (95% CI)^b^	
			Distribution	Scored^c^
**Fruits**				
	**Always**	12428	**71.5 (69.3,73.7)**	
	**Most of the time**	3566	**18.3 (16.8, 19.9)**	**89.8 (88.7, 91.0)**
	Sometimes	1676	8.2 (7.2, 9.2)	
	Rarely	324	1.8 (1.3, 2.3)	10.2 (9.0, 11.3)
	Never	37	0.2 (0.1, 0.3)	
**Dark green vegetables** ^d^				
	**Always**	10543	**58.5 (56.5, 60.5)**	
	**Most of the time**	4031	**22.8 (21.2, 24.5)**	**81.3 (79.8, 82.8)**
	Sometimes	2602	13.7 (12.2, 15.2)	
	Rarely	591	3.4 (2.9, 3.9)	18.7 (17.2, 20.2)
	Never	264	1.6 (1.1, 2.1)	
**Salty snacks**				
	Always	7385	45.2 (42.7, 47.7)	
	Most of the time	3319	19.6 (18.5, 20.7)	88.3 (87.1, 89.5)
	Sometimes	4731	23.5 (21.5, 25.5)	
	**Rarely**	1988	**9.2 (8.1, 10.2)**	
	**Never**	608	**2.5 (2.1, 2.9)**	**11.7 (10.5, 12.9)**
**Sugar-sweetened beverages**				
	Always	7453	42.8 (40.2, 45.4)	
	Most of the time	2662	14.2 (13.0, 15.4)	74.0 (72.0, 76.0)
	Sometimes	3514	16.9 (15.4, 18.5)	
	**Rarely**	2361	**13.7 (12.2, 15.2)**	
	**Never**	2041	**12.3 (11.1, 13.4)**	**26.0 (24.0, 28.0)**
**Aggregate healthy food availability score** ^e^				
	4 (high)	943	4.7 (4.0, 5.5)	
	3	3760	21.6 (20.1, 23.2)	
	2	9975	55.4 (53.7, 57.1)	
	1	2564	14.1 (12.6, 15.6)	
	0 (low)	789	4.1 (3.3, 4.9)	

^a^ Unweighted n

^b^ Percentages weighted and may not add up to 100% due to rounding; 95% CIs account for complex survey design

^c^ Binary variables coded as 1 for healthier (bolded) response and 0 for less healthy (not bolded) response.

^d^ Dark green vegetables were bok choy, broccoli, collard greens, dark green leafy lettuce, kale, mesclun, mustard greens, romaine lettuce, turnip greens, spinach, watercress.

^e^ Aggregate healthy food availability score is the sum of the binary responses.

A healthier pattern of household food availability was associated with greater frequency of family meals at home. The odds (95% CI) of having more family meals per week among those who always or most of the time had fruits in the home were 2.08 (1.80 to 2.41) times those of individuals in households who sometimes, rarely, or never had fruits available. This odds ratio (95% CI) for dark green vegetables was 1.66 (1.37 to 2.01). Individuals living in households with limited availability of salty snacks and sugar-sweetened beverages were also more likely to have more frequent family meals. Rarely or never having salty snacks available in the home was associated with a 25% higher odds (95% CI, 1.02 to 1.55) of having more frequent family meals (P = 0.03), and never or rarely having sugar-sweetened beverages available was associated with a 54% higher odds (95% CI, 1.32 to1.80) for more frequent family meals (P<0.0001).

**Fig 1:**
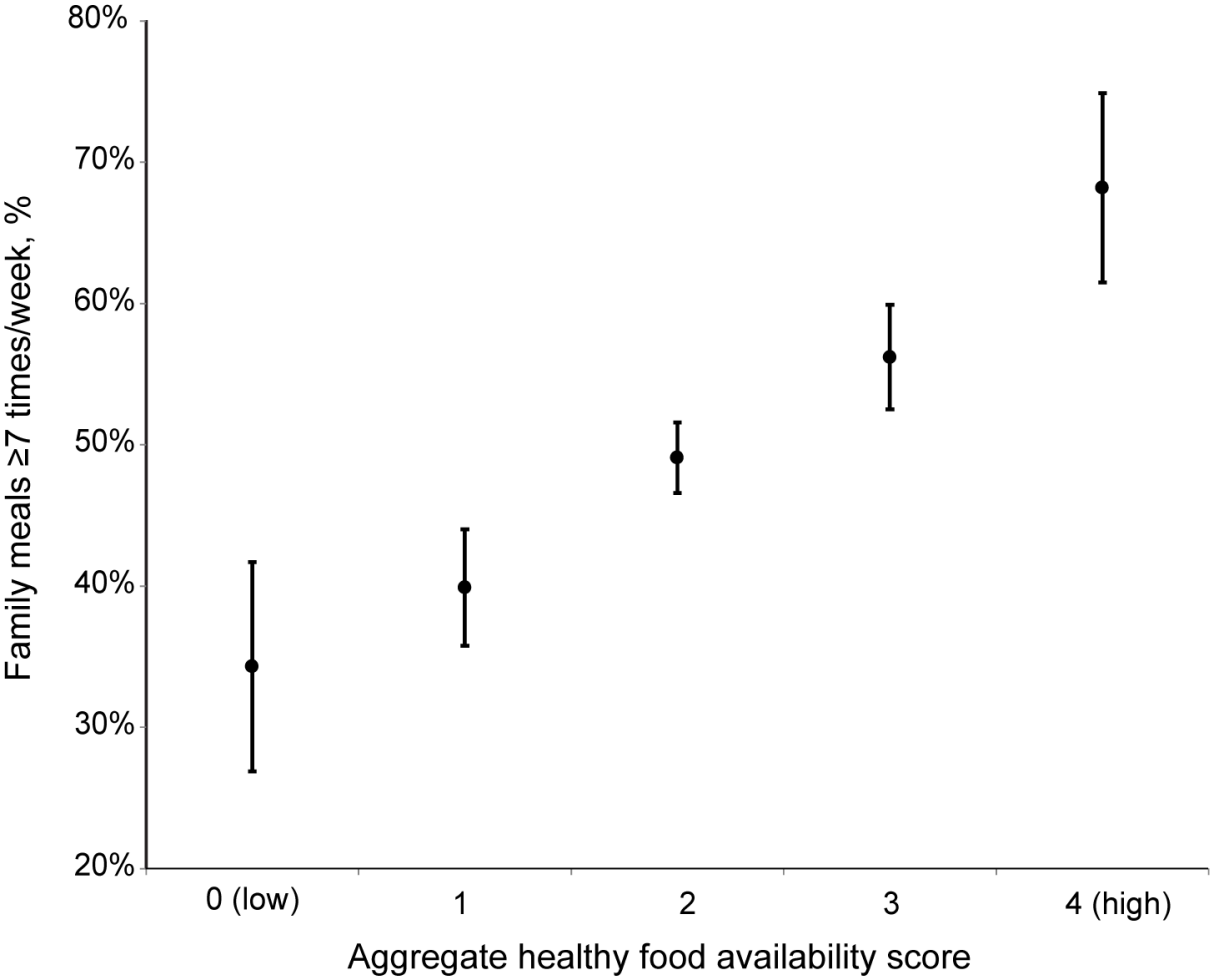
Prevalence of family meals ≥7 times/week by household food availability: U.S. multi-person households, NHANES 2007–10. Aggregate healthy food availability score constructed as sum of 4 binary variables indicating frequent availability of fruits and dark green vegetables and infrequent availability of salty snacks and sugar-sweetened beverages. A score of 4 indicates that the household always or most of the time had fruits and dark green vegetables available, and rarely or never had salty snacks or sugar- sweetened beverages available. Error bars are 95% confidence intervals (CI). Sample sizes at each level of food availability score (low to high) are 789; 2564; 9975; 3760; 943. Percentages are weighted and 95% CI account for the complex sample design.

The aggregate healthy food availability score was associated with a stepwise increase in the prevalence of having ≥7 family meals per week; 68.2% of individuals living in households with the healthiest food availability pattern and 34.3% of those with the least healthy pattern had ≥7 family meals per week (Fig 1). Each one unit increase in aggregate healthy food availability score was associated with an odds ratio (95% CI) for more frequent family meals of 1.44 (1.33 to 1.55) (Table 3).

**Table 3:** Household food availability and family meal frequency in U.S. multi-person households: NHANES 2007–10.

Household food availability		Frequency of family meals at home in the past week			P value	Odds ratio^b^
		Percentage (95% CI)^a^				(95% CI)
Type	Availability	0–2 meals/week	3–6 meals/week	≥7 meals/week		
**Fruits**						
	Always or most of the time	16.3 (15.0, 17.7)	32.5 (31.1, 34.0)	51.1 (49.2, 53.0)	<0.0001	2.08 (1.80, 2.41)
	Sometimes, rarely, or never	32.3 (28.2, 36.5)	31.3 (25.8, 36.8)	36.4 (32.3, 40.4)		1.0 (reference)
**Dark green vegetables**						
	Always or most of the time	16.1 (14.4, 17.9)	32.1 (30.3, 34.0)	51.7 (49.4, 54.0)	<0.0001	1.66 (1.37, 2.01)
	Sometimes, rarely, or never	25.9 (22.6, 29.1)	33.7 (29.6, 37.8)	40.4 (36.2, 44.7)		1.0 (reference)
**Salty snacks**						
	Rarely or never	18.8 (15.2, 22.3)	24.7 (21.1, 28.3)	56.5 (51.3, 61.8)	0.03	1.25 (1.02, 1.55)
	Always, most of the time, or sometimes	17.9 (16.4, 19.3)	33.4 (31.8, 35.1)	48.7 (46.8, 50.6)		1.0 (reference)
**Sugar-sweetened beverages**						
	Rarely or never	12.6 (10.4, 14.8)	30.4 (27.4, 33.5)	57.0 (53.2, 60.8)	<0.0001	1.54 (1.32, 1.80)
	Always, most of the time, or sometimes	19.8 (18.2, 21.5)	33.1 (31.2, 35.0)	47.0 (45.1, 49.0)		1.0 (reference)
**Aggregate healthy food availability score**						
	4 (high)	11.8 (7.5, 16.0)	20.0 (14.4, 25.7)	68.2 (61.6, 74.9)		
	3	11.6 (9.3, 14.0)	32.2 (29.3, 35.1)	56.2 (52.4, 59.9)		
	2	17.8 (15.7, 19.9)	33.1 (30.7, 35.5)	49.1 (46.6, 51.6)	<0.0001	1.44^c^ (1.33, 1.55)
	1	26.4 (23.3, 29.5)	33.7 (29.4, 37.9)	39.9 (35.9, 44.0)		
	0 (low)	31.3 (24.7, 38.0)	34.4 (25.0, 43.7)	34.3 (26.9, 41.7)		

^a^ Percentages are weighted and 95% CI account for the complex sample design.

^b^ Odds ratio from cumulative logistic (proportional odds) models; outcome = frequency of family meals (3-level variable), predictor = food availability (binary variable with less healthy response as the reference category).

^c^ Odds ratio is for a 1 unit difference (higher) aggregate healthy food availability score.

## Discussion

We report U.S. population estimates for family meal frequency at home in multi-person households during the period of 2007–2010. These analyses of the large and nationally-representative National Health and Nutrition Examination Survey add to a limited, but growing, epidemiologic literature on eating environments in all types of U.S. households. Until recently, most studies of family meals have been focused on the experiences of children and adolescents [4], with some research on young adults [12,13] and parents [7]. However, in a large representative survey of Ohio adults conducted in 2012, the frequency of family meals was similar among adults who did and did not have minor children in the household; in fact, family meals were slightly more prevalent in the households that did *not* include children [8]. Thus, it is increasingly recognized that family meals are important across the life course [4], and the study of family meal patterns, benefits, and challenges should not be restricted to families with children [6,8,13,24,25].

Sobal and Hanson described family meal frequency in a national sample of 1000 U.S. adults of whom 88% did not live alone [9]. They reported that 52.6% of U.S. adults ate family meals 7 or more times per week, 32.7% did so 3 to 6 times per week, and 14.6% 0 to 2 times per week [9]. A smaller study the following year asked about frequency of family *dinner* and estimated that 46% of the population ate dinner with their family every day [6]. Our U.S. prevalence estimates from NHANES 2007–10 are similar to these prior reports [6, 9]. We found a somewhat higher prevalence (18.0%) of having few family meals per week and a slightly lower prevalence (49.6%) of having ≥7 family meals per week. However, differences in the survey methodology and sampling strategy limit direct comparison. Our analyses benefit from the large sample size, methodological rigor and standardization used in the design and conduct of NHANES [15], and our estimates are generalizable to the U.S. population living in multi-person households.

Having frequent family meals is common among U.S. households across all sociodemographic characteristics. We found that married/partnered households were more likely to have frequent family meals, as were older respondents, those who had less education, and those with lower household income; this is consistent with evidence from other studies [6,8,9,25]. Employment status and time availability may offer possible explanations for this pattern. We did not have information in NHANES about the employment status of the household respondent, but others have found that family meals are more frequent in households in which one or more adult is not employed either because of retirement, disability, unemployment, or being a “stay-at-home parent” [8,26]. Time constraints associated with employment, particularly when all adults in the household are employed, may make it more difficult for households to have family meals at home [1,27]. Households in which the respondent was born outside the U.S. were much more likely to have ≥7 family meals at home per week (60% of households headed by someone born outside the U.S. compared to 47% of households headed by an adult born in the U.S.). A number of studies have suggested that nutritional and health related-behaviors of individuals born in other countries are negatively impacted by living in the U.S. [28,29]. The causes for this are likely to be complex and nuanced [27,30].

NHANES asked about the prevalence of meals eaten as a family at home in the past week. Virudachalam and colleagues [19] reported on the prevalence and sociodemographic correlates of cooking dinner at home in U.S. households in 2007–2008 based on analyses of NHANES. In line with our findings for the frequency of family meals at home, they found that cooking dinner at home was more common among older households, those with low levels of income or education, and households headed by someone who was born outside the U.S. [19]. Having frequent family meals and cooking at home are generally considered “healthy” behaviors. As also noted by Virudachalam [19], our analyses demonstrate a higher frequency of these healthy behaviors among individuals who have low levels of income and education.

Many studies have reported that children and adolescents who eat frequent family meals have more nutritious diets [4,10,11]. Less is known about the correlates of family meals in adults, but some evidence links higher fruit and vegetable consumption to family meal frequency among parents of adolescents [7] and among young adults [24]. We found a stepwise relationship between our aggregate healthy food availability score and increasing family meal frequency. Each of the four components of this score (high availability of fruits and dark green vegetables; low availability of salty snacks and sugar-sweetened beverages) were individually related to family meal frequency and the association was stronger when these four aspects of food availability were combined. Masters and colleagues reported on home food availability for 6–19 year-old youth in NHANES 2007–10 [31]. They found: 1) fruit was more likely to always be available in high-income households and that this was true across race/ethnicity groups, 2) availability of dark green vegetables was not strongly associated with income or race/ethnicity, 3) salty snacks were more likely to always be available for non-Hispanic white youth, particularly those living in high-income households, and 4) across income levels, Hispanic youth were less likely than non-Hispanic white youth to always have sugar-sweetened beverages available [31]. They did not examine how household food availability was related to family meals.

Family meals require planning to orchestrate, and if someone in the household is investing the time needed to have family meals, they are also likely to be thinking about what foods to purchase for the household. Individuals living in households in which competing demands make having family meals difficult (e.g., changing employment schedules or children’s activities) may not prioritize food shopping and meal planning. Consistent with this, in a study of 277 parents of adolescents in Minnesota, >45% of respondents reported that they “often don’t think about what to have for dinner until right before dinner” and that family work and activity schedules “often make it difficult to have family meals together” [32]. Thus, there is likely to be a positive association between the healthfulness of foods available in the home and the frequency of family meals which is what we observed in our analyses. In the context of the current U.S. food environment, it is challenging to eat healthfully without planning and organization. Many of the sociodemographic characteristics that were associated with greater family meal frequency (e.g., older age) may indicate that the household contains at least one person who has the time available to plan and prepare meals, but we were not able to assess this aspect of household composition directly. For individuals who are not thinking about their food choices and who have limited time, the foods they are most likely to encounter (e.g., snack foods or fast foods) are often the least healthy.

Our results should be interpreted in the context of their limitations. NHANES is a cross-sectional study and our analyses are descriptive; thus, no causal associations should be inferred. We were unable to characterize the ages and relationships of individuals within households. In addition, multiple people may be sampled from within a household [15,33]. This within-household clustering is accounted for by the NHANES survey weights and not recoverable in the released data. An important implication is that although an adult reported on the frequency of family meals and food availability for their household, our prevalence estimates apply to the full population of individuals (including children) living in U.S. multi-person households. A single question assessed the number of meals that most of the family ate together at home during the past 7 days. We do not know the cognitive process [34] that respondents used to answer this question but it is likely that many estimated based on their typical pattern of behavior. The family meal question asked about *most* rather than all of the family being present so we may be overestimating the percentage of U.S. individuals having frequent family meals. It is also important to recognize that our categories of family meal frequency (0–2 meals, 3–6 meals, ≥7 meals per week) do not specify the number of *days* per week that the family meals occurred. Although it is likely that those we categorized as having frequent family meals (≥7 meals per week) had a family meal every day, this category could also include respondents with more irregular patterns (e.g., breakfast and dinner as a family on 4 days/week). In the U.S., dinner is the meal most likely to be eaten as a family [26,35], and research on family meals has focused primarily on family dinners [10]. Our estimates are for meals eaten at home and do not include family meals in restaurants, community settings, or friends’ houses. Finally, we do not know what was served or eaten during family meals, nor did we assess whether the food was prepared at home.

Only recently have studies of family meals included households without children, and our report adds to this growing literature. A positive association between frequent family meals and healthful dietary intake has been demonstrated in children and adolescents [4], with some evidence also indicating nutritional benefits of shared meals among young adults [13]. Our research suggests this association may not be limited to young people. Expanding research on the benefits of family meals, and shared meals generally, to include adults who do not have children, could ultimately impact the reach and targeting of public health messages and interventions. Strategies to encourage more healthful eating are needed to reduce the health burdens of obesity and diet-related chronic disease, but more research is necessary to understand why eating with others appears to be associated with healthier patterns of household food availability. The results of which are likely to open new avenues of inquiry and lead to more effective interventions, programs, and policies. For example, the social context of eating can impact people’s objective and subjective experiences of food consumption [36]. The public health implications of eating with others have yet to be fully understood, and this is particularly true for adults who are not living with minor children. Nevertheless, with awareness that mealtimes are complex [1,2], nutrition and health professionals can be encouraged to support all households, regardless of their configuration, in efforts to eat more meals together as a family.
